# Anti-Inflammatory and Anti-Adipocyte Dysfunction Effects of *Ficus lindsayana* Latex and Root Extracts

**DOI:** 10.3390/ph17030287

**Published:** 2024-02-23

**Authors:** Jirarat Karinchai, Pensiri Buacheen, Daraphan Rodthayoy, Tanpitcha Yodweerapong, Arisa Imsumran, Ariyaphong Wongnoppavich, Bhanumas Chantarasuwan, Uthaiwan Suttisansanee, Piya Temviriyanukul, Pornsiri Pitchakarn

**Affiliations:** 1 Department of Biochemistry, Faculty of Medicine, Chiang Mai University, Muang Chiang Mai, Chiang Mai 50200, Thailand; jirarat.ka@cmu.ac.th (J.K.); pensiri.bc@gmail.com (P.B.); daraphanr@gmail.com (D.R.); tanpitcha.y@hotmail.com (T.Y.); arisa.bonness@cmu.ac.th (A.I.); ariyaphong.w@cmu.ac.th (A.W.); 2 Thailand Natural History Museum, National Science Museum, Khlong 5, Khlong Laung, Pathum Thani 12120, Thailand; b.chantarasuwan@gmail.com; 3 Institute of Nutrition, Mahidol University, Salaya, Nakhon Pathom 73170, Thailand; uthaiwan.sut@mahidol.ac.th (U.S.); piya.tem@mahidol.ac.th (P.T.)

**Keywords:** *Ficus lindsayana*, adipogenesis, insulin resistance, chronic inflammation, metabolic diseases

## Abstract

Low-grade chronic inflammation and adipocyte dysfunction are prominent risk factors of insulin resistance and type 2 diabetes mellitus (T2DM) in obesity. Thus, prevention of inflammation and adipocyte dysfunction could be one possible approach to mitigate T2DM development. Several *Ficus* species have been used in traditional medicine for ameliorating inflammation and T2DM. Our previous studies reported biological effects of *Ficus lindsayana* including antioxidant, anti-cancer, and anti-α-glucosidase activities. Further, this study therefore investigated whether *F. lindsayana* latex (FLLE) and root (FLRE) extracts inhibit inflammation-stimulated insulin resistance in adipocytes and inflammation in macrophages. FLLE and FLRE (200 µg/mL) had no significant cytotoxicity for macrophages, adipocytes, and blood cells (PBMCs and RBCs). FLRE had a total flavonoid content about three times higher than FLLE, while both had similar levels of total phenolic content. FLRE showed higher abilities than FLLE in suppressing inflammation in both macrophages and adipocytes and reversing the inflammation-induced insulin resistance in adipocytes. In TNF-α-induced adipocytes, FLRE significantly improved insulin-induced glucose uptake and insulin-suppressed lipolysis, while FLLE only significantly improved glucose uptake. Moreover, FLRE and FLLE remarkably reduced chemoattractant (MCP-1) but improved adipogenic (PPARγ and CEBPα) gene expression, leading to the promotion of adipogenesis and the suppression of insulin resistance. In LPS-induced macrophages, FLRE, but not FLLE, significantly inhibited LPS-induced NO production. Moreover, FLRE significantly reduced LPS-stimulated iNOS, COX-2, IL-1β, IL-6, and TNF-α gene expression. These results may provide the potential data for the development of this plant, especially the root part, as an alternative medicine, functional ingredient, or food supplement for the prevention of inflammation and obesity-associated insulin resistance, as well as T2DM.

## 1. Introduction

Insulin resistance generally refers to a condition in which the body does not respond properly to insulin, affecting glucose transport and metabolism in adipocytes and skeletal muscle, as well as decreasing the ability to suppress glucose production in the liver. Normally, insulin promotes the development of preadipocytes into adipocytes and stimulates glucose transport and facilitates the production of triglycerides (lipogenesis) in mature adipocytes, while also preventing the breakdown of triglycerides (lipolysis). Recently, it has been well-established that adipocyte dysfunction plays a central role in linking insulin resistance to obesity. An imbalance of hypertrophy/hyperplasia, where adipogenesis is suppressed, can cause the over-expansion of adipocytes (hypertrophic adipocytes). The adipocyte hypertrophy prevalence induces hypoxia and cellular stress that promote chronic inflammation by secreted chemokines, especially monocyte chemoattractant protein-1 (MCP-1) from adipocytes, leading to the recruitment of pro-inflammatory macrophages into the adipose tissue [1]. The macrophages then generate several pro-inflammatory cytokines, including tumor necrotic factor α (TNF-α), interleukin-1β (IL-1β), and interleukin-6 (IL-6) [2]. The increase in these cytokines causes adipocyte dysfunction and inhibits insulin signaling, leading to insulin resistance [1,3,4]. Thus, obesity could cause chronic low-grade systemic and local inflammation that leads to the emergence of insulin resistance and type 2 diabetes mellitus (T2DM).

Chronic inflammation is a prolonged inflammatory response that follows an acute inflammation or long-term exposure to low-grade inflammation. It involves a progressive change in the type of cells present at the inflammatory site. In chronic inflammation conditions, many chemotactic and activating factors are continually released from immune cells to stimulate immune and stromal cells. A variety of inflammatory cytokines, such as TNF-α, IL-1β, IL-6, inducible nitric oxide synthase (iNOS), and cyclooxygenase 2 (COX-2) secreted from the macrophages are further involved in various inflammation-related diseases [5]. Moreover, chronic inflammation is usually associated with free radical damage and oxidative stress, which can cause cancer by mutation and DNA damage [6,7]. Therefore, the prevention of inflammation could protect people from inflammation-induced disorders including insulin resistance in obesity and T2DM. Anti-inflammation agents have been demonstrated to improve the sensitivity and secretion of insulin in patients with T2DM [8,9]. However, these medications, especially non-steroidal anti-inflammatory drugs (NSAIDs), which block the COX enzyme and reduce prostaglandins [10] usually cause multiple adverse effects on the gastrointestinal tract, cardiovascular, and kidney function [11]. So, the usage of existing natural products for inflammation prevention is one of the alternative ways to treat inflammation-induced chronic diseases. Many phytochemicals have been reported for their pharmacological activities such as anti-diabetes type 2 [12], anti-cancer [13], and anti-arthritic properties [14] via mechanisms involving the suppression of oxidative stress, inflammation, and tissue damage [15].

Plants in the *Ficus* genus include over 800 species that have been consumed as food and utilized for medicinal properties in Ayurvedic and traditional Chinese medicine for centuries [16,17]. *Ficus* spp. are distributed in tropical and subtropical regions of India, China, Sri Lanka, Australia, and Myanmar. Various parts, such as tree bark, fruits, latex, leaves, roots, and twigs of *Ficus* spp. have been reported to be used in traditional medicine for treating conditions like diarrhea, stomach problems, cancer, inflammation and diabetes [18,19,20]. For example, *Ficus carica* Linn has antioxidant and antimicrobial activities [21] and remarkable curative effects for chronic diseases [19]. *Ficus sycomorus* Linn (bark), *Ficus racemosa* Linn (bark), *Ficus lutea* Vahl (leaf), and *Ficus deltoidei* Jack(fruit) exhibit anti-diabetic effects [19,22,23,24]. Moreover, flavonoids in the *Ficus* sp. extracts are associated with their blood glucose-reducing activity, anti-cancer, antioxidant, antihistaminic, and anti-inflammation [20,25,26]. 

*Ficus lindsayana* (FL) Beentje, previously named *Ficus dubia* Wall. ex King [27] is another species found in the tropical evergreen rain forests of Southern Thailand, Malaysia, and Brunei [28] (The plant name has been checked with WFO (http://www.worldfloraonline.org/taxon/wfo-1000027124, accessed on 25 December 2023)). The tree can grow to be 30–35 m tall, with auxiliary aerial roots that extend to the ground from branches or the trunk. Due to its unique red latex after exposure to the air, local people believe that this plant may offer health benefits, similar to Dragon’s blood, an acclaimed traditional medicine obtained from different plants such as *Croton* spp., *Daemonorops* spp., *Dracaena* spp., and *Pterocarpus* spp. [29]. Previous studies reported several biological activities of various parts of FL. The extract from stems and twigs showed anti-HIV and anti-bacterial activities [30]. The root and latex extracts of FL exhibited strong antioxidant activity correlated with the high amount of total phenolics and flavonoids [31,32]. In addition, the root ethanolic extract displays potent antidiabetic effects through the inhibition of α-glucosidase, one of the drug targets for diabetes, and anticancer activity against lung- and ovarian-cancer cell lines with genome-safe [31]. Moreover, it was found that the latex extract has the potential for the prevention and treatment of human colorectal cancer through the regulation of the inflammatory pathway [33,34]. Although FL has been reported to have the potential against inflammation and diabetes, there is no scientific information of its bioactive compounds and biological activity on inflammation-associated adipocyte dysfunction, which is a major cause of insulin resistance and diabetes (T2DM).

In this study, we determined whether *Ficus lindsayana* latex and root extracts affect adipogenesis, insulin resistance in 3T3-L1 adipocytes, and inflammation in RAW 264.7 macrophages. The knowledge gained from this study will support evidence of the health benefits of this plant for further development as an alternative medicine, functional ingredient, or food supplement in preventing obesity-related and inflammation-involved metabolic disorders, especially insulin resistance and T2DM.

## 2. Results

### 2.1. Phenolic and Flavonoid Composition of the FLLE and FLRE

The yield of FLLE and FLRE was 47% and 5%, respectively. The total phenolic content of FLRE and FLLE was 208.31 ± 9.90 and 248.53 ± 1.46 mg GAE/g extract, respectively (Table 1). The total flavonoid content of FLRE and FLLE was 157.33 ± 1.36 and 55.78 ± 4.00 mg CE/g extract, respectively (Table 1). The result showed that FLLE contained a higher amount of phenolic content, whereas FLRE contained a higher level of total flavonoid content than the other extract.

Compared to the known standard phenolics and flavonoids, chlorogenic acids (CGAs) and apigenin were detectable in FLRE, while only CGAs were detectable in FLLE by HPLC analysis (Table 2). Determination of chlorogenic derivatives including 3-, 4-, and 5-CQA showed 3-CQA was the most abundant in FLLE and FLRE. The content of CGAs and the derivatives were much greater in FLLE than in FLRE.

Consistent with the results from the HPLC analysis, the LC-MS analysis revealed that CGAs found in FLLE were higher than in FLRE. Moreover, caffeic acid was detectable in FLLE and FLRE. Apigenin, genistein, 3,4-dihydroxybenzoic acid, naringenin, luteolin, and gallic acid were detected in FLRE, but not in FLLE (Table 3). These results demonstrated that FLLE and FLRE contained different phytochemical patterns which may influence their biological activities.

### 2.2. DPPH^•^ and ABTS^•+^ Scavenging Activities of FLLE and FLRE

This experiment investigated the antioxidant activity of the extracts using DPPH and ABTS assays. The result showed that FLLE and FLRE significantly scavenged DPPH^•^ and ABTS^•+^ free radicals, as shown in Table 1. The results from the DPPH assay showed that SC50 of FLLE and FLRE was 210.59 ± 6.13 and 83.12 ± 8.86 µg/mL, respectively, while the SC50 of FLLE and FLRE was 33.59 ± 6.26 and 9.44 ± 3.26 µg/mL, respectively, determined by the ABTS assay. In addition, the SC50 of the extracts determined by the ABTS assay was lower than that of the DPPH assay, possibly due to the solvent used in each assay (DPPH; EtOH, ABTS; water). The major antioxidants in the extracts are probably water-soluble compounds.

### 2.3. Cytotoxicity of FLLE and FLRE on RAW 264.7 Macrophages, Human PBMCs, Human RBCs, and Mature 3T3-L1 Adipocytes

**Figure 1:**
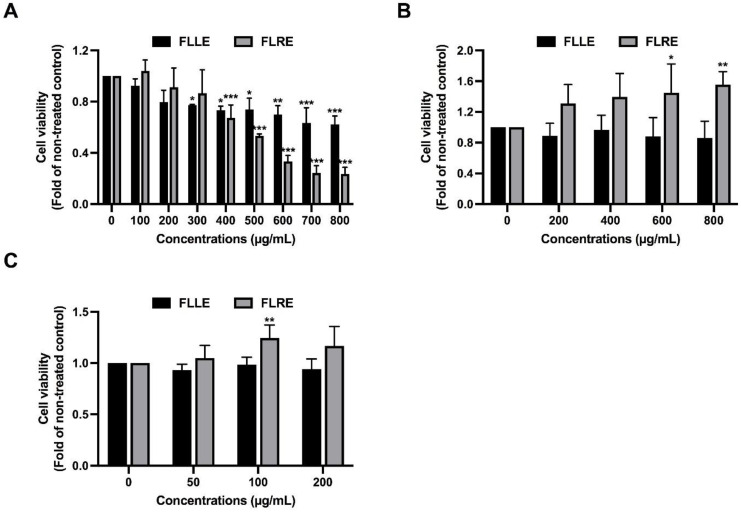
Cytotoxicity effect of FLLE and FLRE. Cell viability of RAW 264.7 macrophages (**A**), normal human PBMCs (**B**), and 3T3-L1 mature adipocytes (**C**). RAW 264.7 cells and PBMCs were treated with various concentrations of the extracts (0–800 µg/mL) for 48 h. Cell viability was determined by MTT assay. 3T3-L1 mature adipocytes were treated with 0–200 µg/mL of the extracts for 48 h, and cell viability was then examined by SRB assay. Each value represents mean ± SD (*n* = 3) * p < 0.05, ** p < 0.01 and *** p < 0.001 vs. non-treated control.

FLLE and FLRE at doses up to 200 µg/mL slightly decreased the cell viability of RAW 264.7 macrophages (Figure 1A). The inhibitory concentration of 20 (IC20) of FLLE and FLRE was 244 and 331 µg/mL, respectively. The half-maximal inhibitory concentration (IC50) of FLLE and FLRE was >800 and 506 µg/mL, respectively.

Figure 1B shows that FLLE and FLRE at doses up to 800 µg/mL did not show any toxicity to human PBMCs. Table 4 shows that FLRE at the dose of up to 400 µg/mL induced hemolysis at less than 10%, while 600 and 800 µg/mL of the extract caused hemolysis of approximately 10–13%. FLLE at the dose of up to 800 µg/mL slightly induced hemolysis of approximately 3–6% when compared with 0.05% triton x-100, which was used as a positive control (100% hemolysis). Normal saline was used as a negative control (0% hemolysis). Thus, the results can be interpreted as showing that FLRE at the concentration of up to 400 µg/mL was not toxic to human RBCs, while the concentration of up to 800 µg/mL slightly induced hemolysis. FLLE (at up to 800 µg/mL) was a non-hemolytic induction agent.

In mature 3T3-L1 adipocytes, the concentration of extracts at 200 µg/mL did not show any significant cytotoxicity, which is shown in Figure 1C.

These results suggested that FLRE at high concentration was more cytotoxic to the cells than FLLE. The non-toxic concentrations (0–200 µg/mL) were used for further experiments.

### 2.4. Effect of FLLE and FLRE on TNF-α-Induced Insulin Resistance and Adipogenesis in 3T3-L1 Cells

**Figure 2:**
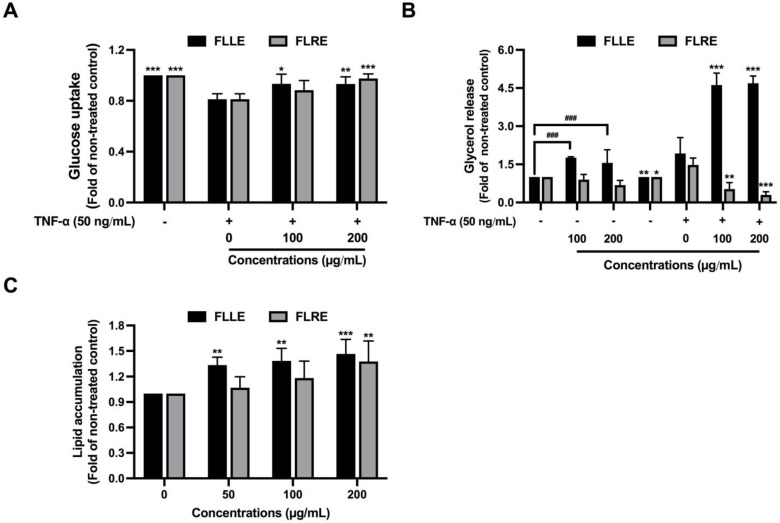
Effect of FLLE and FLRE on TNF–α–induced insulin resistance in 3T3–L1 adipocytes. Anti–insulin resistance effects of the extracts on glucose uptake (**A**) and lipolysis (**B**). Mature 3T3––L1 adipocytes were co–treated with TNF–α (50 ng/mL) and various concentrations of FLLE or FLRE for 24 h. The treated cells were subjected to determined insulin-stimulated glucose uptake using 2–NBDG, a glucose fluorescence analog. The conditioned medium was collected to measure the glycerol level indicating lipolysis activity using a glycerol detection kit. The effect of FLLE and FLRE on adipogenesis in 3T3–L1 adipocyte is shown in (**C**). To determine the effect of FLLE and FLRE on lipid accumulation in 3T3–L1 adipocytes, cells were treated with the extracts during cell differentiation for 12 days. Lipid accumulation was performed using Oil Red O assay. Each value represents mean ± SD (*n* = 3) * p < 0.05, ** p < 0.01, and *** p < 0.001 vs. TNF–α–treated group (**A**,**B**) or vs. non–treated control (**C**), *### p <* 0.001 vs. non-treated control.

To evaluate the anti-insulin resistance activity of FLLE and FLRE, insulin-induced glucose uptake ability was measured in TNF-α-induced 3T3-L1 adipocytes. Treatment of the cells with TNF-α 50 ng/mL significantly decreased glucose uptake ability induced by insulin at approximately 19% when compared with the control group (Figure 2A). In contrast, co-treatment of FLLE (100 and 200 µg/mL) and FLRE (200 µg/mL) in TNF-α-treated cells at 100 and 200 µg/mL significantly enhanced the insulin-stimulated glucose uptake (Figure 2A).

To confirm insulin resistance in adipocytes, lipolysis was also determined by measuring glycerol release. TNF-α 50 ng/mL treatment significantly increased the glycerol level in the culture supernatant by 20%, even in the presence of insulin. FLRE (100, 200 µg/mL) significantly suppressed the glycerol release; on the other hand, FLLE (100, 200 µg/mL) dramatically induced the release in TNFα-treated adipocytes (Figure 2B). Treatment of FLLE in the absence of TNF-α significantly induced the release of glycerol, suggesting the lipolytic effect of this extract.

FLLE (50, 100, 200 µg/mL) and FLRE (200 µg/mL) treatment during differentiation for 12 days significantly increased lipid accumulation in the adipocytes (Figure 2C), suggesting that the extracts significantly promoted adipogenesis, which may either lead to the expansion of adipose tissue (induce obesity) or the prevention of insulin resistance (improved adipocyte function) [35,36].

### 2.5. Effect of FLLE and FLRE on the mRNA Expression of Inflammatory-Associated and Adipogenesis-Related Molecules in TNF-α-Induced 3T3-L1 Adipocytes

**Figure 3:**
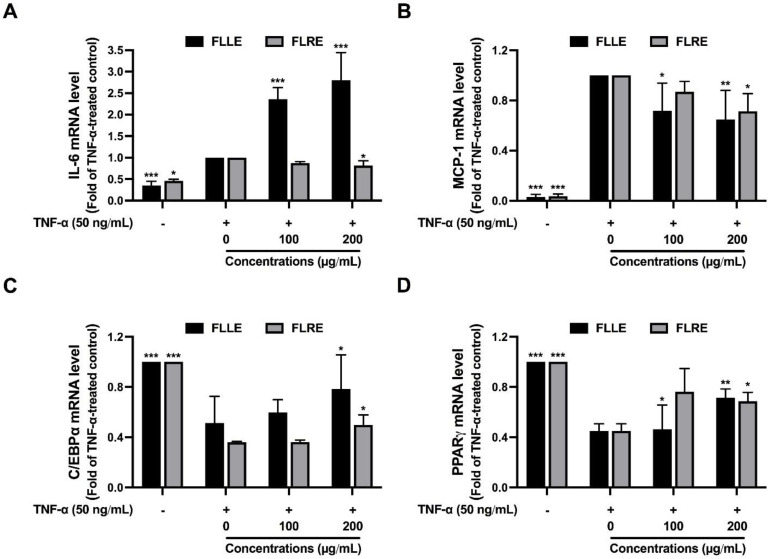
Effect of FLLE and FLRE on TNF–α–induced adipocyte inflammation and dysfunction. Effect of FLLE and FLRE on the mRNA expression of an inflammatory–associated molecule including IL–6 (**A**) and MCP–1 (**B**), and adipogenesis–related molecules including CEBPα (**C**) and PPARγ (**D**). Total RNA was extracted from the treated cells and subjected to RT–qPCR with specific primers for IL–6, MCP–1, CEBPα, and PPARγ. Each value represents mean ± SD (*n* = 3) * p < 0.05, ** p < 0.01, and *** p < 0.001 vs. TNF–α–treated group.

The next experiments investigated the effect of FLLE and FLRE on adipocyte inflammation and dysfunction. MCP-1 and IL-6 mRNA expressions were measured to observe the inflammatory condition in the cells. TNFα-treated 3T3-L1 adipocytes showed the significant upregulation of IL-6 and MCP-1 mRNA, whereas FLRE (200 µg/mL) significantly suppressed the TNFα-stimulated IL-6 and MCP-1 mRNA expressions (Figure 3A,B). FLLE (100, 200 µg/mL) significantly reduced MCP-1 mRNA expression but markedly induced IL-6 mRNA expression in the TNFα-treated cells (Figure 3A,B). The result suggested that FLRE may inhibit the transcription of MCP-1 and IL-6, while FLLE may inhibit the transcription and reduce the protein level of MCP-1 but induce mRNA and protein levels of IL-6. 

We further determined the expression of PPARγ and CEBPα, adipogenic genes which normally express in mature adipocytes, to observe adipocyte dysfunction in TNFα-treated 3T3-L1. Both CEBPα and PPARγ mRNA expressions were significantly suppressed by TNFα treatment. Interestingly, FLLE and FLRE (200 µg/mL) significantly improved CEBPα and PPARγ mRNA expressions when compared with the TNFα-treated group (Figure 3C,D).

These results suggested that TNFα induced inflammation and dysfunction of adipocytes, whereas FLLE and FLRE abrogated these effects. It could be confirmed that FLLE and FLRE rescued insulin resistance via adipogenesis induction and inflammation suppression, leading to an enhancement of adipocyte function and insulin sensitivity.

### 2.6. Anti-Inflammatory Activity of FLLE and FLRE in LPS-Stimulated RAW 264.7 Macrophages

**Figure 4:**
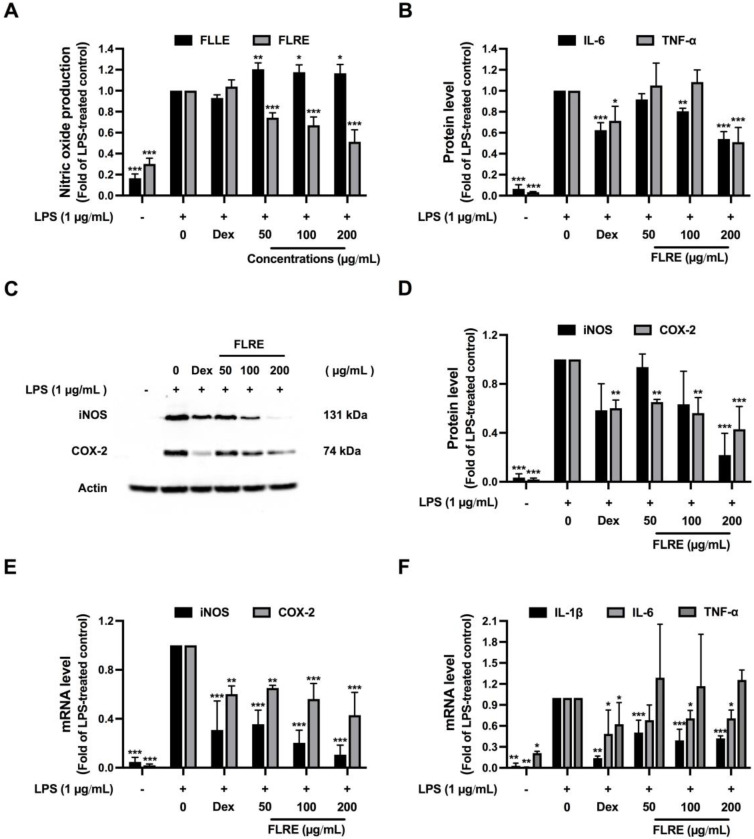
Anti–inflammation activity of FLLE and FLRE in LPS–stimulated RAW 264.7 macrophage. Effect of the extracts on NO production (**A**). Effect of FLRE on LPS–stimulated pro–inflammatory cytokine secretions including IL–6 and TNF–α (**B**). Effect of FLRE on inflammatory key enzymes protein level and mRNA expression. Immunoblotting of iNOS and COX–2 protein (**C**), the quantitative protein level of iNOS and COX–2 normalized with β–actin (**D**). iNOS and COX–2 mRNA expression by qRT–PCR (**E**). Effect of FLRE on pro-inflammatory cytokine mRNA expression including IL–1β, IL–6, and TNF–α (**F**). RAW 264.7 macrophages were pre–treated with FLRE or FLLE (0–200 µg/mL) for 1 h followed by 1 µg/mL LPS for 24 h. The supernatant was collected to examine the nitrite level by Griess reagent assay and pro–inflammatory cytokine secretion level ELISA. The treated cells were collected to determine protein and mRNA expression of iNOS, and COX–2 by Western blotting and RT–qPCR, respectively. Each value represents mean ± SD (*n* = 3), * p < 0.05, ** p < 0.01 and *** p < 0.001 vs. LPS-treated group.

As an indicator of an inflammatory response, LPS (1 µg/mL) significantly induced NO production in macrophages as measured by the accumulation of nitrite level in the culture supernatant. The treatment of FLRE significantly reduced the NO level in a dose-dependent manner, whereas FLLE significantly induced the NO level in a dose-dependent manner (Figure 4A). Since FLRE also contained higher flavonoid content and exhibited higher antioxidant efficacy, we further investigated only FLRE on the anti-inflammatory mechanism.

In chronic inflammation, many activating factors and pro-inflammatory cytokines are released from immune cells to stimulate the immune system and further involve various inflammation circumstances. To investigate whether FLRE suppressed pro-inflammatory cytokine secretion from LPS-induced RAW 264.7 macrophages, the protein levels of IL-6 and TNF-α were measured by ELISA. FLRE significantly decreased IL-6 (100 and 200 µg/mL) and TNF-α (200 µg/mL) proteins secreted in the culture supernatant when compared to the LPS-treated group (Figure 4B). The result suggested that the reduction in IL-6 and TNF-α protein secretion may be caused by the inhibition effect of the protein expression or the protein secretion.

LPS stimulates the expression of pro-inflammatory cytokines and mediators such as iNOS and COX-2. We further determined whether FLRE inhibited LPS-stimulated iNOS and COX-2 protein levels. The expression of iNOS and COX-2 proteins was strongly induced in the LPS-treated group but was significantly suppressed after the treatment of 200 µg/mL FLRE (Figure 4C,D). The result suggested that FLRE might inhibit the production of NO via the downregulation of iNOS protein, which may in turn reduce the PGE2 production due to the suppression of COX-2 level. 

The next experiment investigated the effect of FLRE on mRNA expression of iNOS, COX-2, IL-1β, IL-6, and TNF-α by reverse transcription–quantitative real-time PCR. FLRE (50, 100, 200 µg/mL) significantly suppressed iNOS, COX-2, IL-1β and IL-6, and mRNA expressions in LPS-treated RAW 264.7 macrophages (Figure 4E,F), indicating that FLRE may inhibit the transcription of IL-6, leading to the reduction in its secretion. However, TNF-α mRNA expression was not altered (Figure 4F) by FLRE, suggesting that FLRE may inhibit the translation or the secretion of TNF-α in LPS-induced macrophages. Moreover, FLRE decreased iNOS, and COX-2 protein levels via the inhibition of their mRNA expression. It could be summarized that FLRE altered the gene expression of pro-inflammatory cytokines, IL-1β, and IL-6, and inflammation-related proteins iNOS, and COX-2, whereas it probably decreased the TNF-α level at the translation or secretion step.

## 3. Discussion

In this study, our *Ficus lindsayana* root extract (FLRE) had higher total flavonoid content, whereas the latex (FLLE) had a similar amount of total flavonoid content when compared to other previous studies in the same parts of other *Ficus* spp. The total flavonoid content of *Ficus benghalensis* Linn root extracted with ethyl acetate was 6.00 ± 0.55 mg QE/g extract [37]. Meanwhile, the total flavonoid of *Ficus carica* latex extract with water was 43.23 ± 3.35 mg QE/g extract [38]. We found the total flavonoid of FLLE and FLRE was 55.78 ± 4.00 and 157.33 ± 1.36 mg CE/g extract, respectively. FLRE contained a similar amount of phenolic content as FLLE, but showed higher flavonoid content with exhibited higher efficacy of scavenging DPPH^•^ and ABTS^•+^ radicals than FLLE. These data suggest that the antioxidant capacity of the extracts likely depends on their flavonoid contents. The consistency with previous studies of other *Ficus* spp. showed that the flavonoid contents of *Ficus pyriformis* Hook. & Arn. [39] and *Ficus carica* [40] were related to their efficiency in scavenging free radicals and suppressing NO production, a sign of inflammation. Phytochemical analysis revealed that CQA and caffeic acid content found in FLLE was higher than in FLRE, while apigenin, genistein, 3,4-dihydroxybenzoic acid, naringenin, luteolin, and gallic acid were detectable in FLRE, but not in FLLE. 

Chlorogenic acids (CGAs) are ester forms of caffeic acid conjugated with quinic acids. Numerous studies showed several biological properties including antioxidant and anti-inflammation, anti-insulin resistance, and anti-obesity of CGAs, caffeic acid, and also their derivatives through the reduction in iNOS and COX-2 expressions, and also diminished pro-inflammatory cytokine including IL-1β and TNF-α in LPS-induced RAW 264.7 macrophage [41,42,43,44,45]. Likewise, apigenin, genistein, luteolin, and gallic acid have been reported to have an anti-inflammatory effect in LPS-induced RAW 264.7 through the inhibition of NO production, pro-inflammatory cytokines, and the NF-kB pathway. Moreover, apigenin, luteolin, 3,4-dihydroxybenzoic acid, CGAs, and caffeic acid attenuated obesity- and inflammation-induced insulin resistance [42,46,47,48,49,50,51,52,53]. Moreover, previous studies reported that CGAs and gallic acid induce the expression of GLUT4 which promotes the glucose uptake activity in 3T3-L1 cells [52,54]. This evidence can suggest that phenolic and flavonoid compounds found in FLRE and FLLE may play an important role in anti-inflammation and anti-insulin resistance.

Adipocyte inflammation and dysfunction cause abnormal lipid metabolism in adipose tissue, leading to a high blood level of free fatty acids (FFAs) and dysregulation of adipokines [1]. Furthermore, released chemokines and pro-inflammatory cytokines from adipocytes and macrophages constantly induce chronic inflammation surrounding the tissue. Signaling from the cytokines then disturbs insulin signaling by stimulating serine phosphorylation of insulin receptor substrate-1 and 2, leading to the inhibition of insulin receptor autophosphorylation activity. Then, GLUT4 translocation and the blood glucose clearance ability of adipocytes are decreased [1]. Persistence of these conditions can stimulate systemic insulin resistance and beta-cell dysfunction, which would increase the risk of T2DM. It is known that the blockage or reversion of insulin resistance is a promising strategy for the prevention of T2DM and other related metabolic diseases. This study has attempted to prove that FLLE and FLRE prevent inflammation both in adipocytes and macrophages, a major cascade of insulin-resistance progression.

Pro-inflammatory cytokines showed a correlation with insulin resistance in adipocytes, especially TNF-α, a major mediator in the development of chronic inflammation that leads to a reduction in insulin-induced glucose uptake ability [2]. TNF-α initiates an inflammatory signaling cascade via the NF-κB and MAPK pathways, which promotes the release of further inflammatory mediators such as IL-6, IL-10, IL-1β, and MCP-1 [1]. Both FLLE and FLRE treatment could reduce insulin resistance in TNF-α-induced adipocytes, which is consistent with a previous study in which ethanolic and methanolic extracts of *Ficus deltoidei* increased insulin-mediated glucose uptake [55]. FLRE could improve insulin function to induce glucose uptake and suppress lipolysis in the inflammation-induced insulin-resistance model. Although FLLE also enhanced insulin-induced glucose uptake, it failed to improve insulin-suppressed lipolysis in the insulin-resistant cells. FLLE and FLRE may promote insulin function in the insulin-resistant cells via an underlining mechanism(s) by which the adipogenesis is induced and/or the inflammation is suppressed, leading to an improvement in adipocyte function and insulin sensitivity. Furthermore, FLRE significantly decreased MCP-1 and IL-6 mRNA expression. Interestingly, FLLE could suppress only MCP-1, while increasing IL-6 expression by which lipolysis was induced. MCP-1 released from adipocytes is a chemoattractant that activates and recruits macrophages to the area. Moreover, it has a dedifferentiation effect, by altering adipocyte function and metabolism [56]. Previous studies showed that the expression of several adipogenic genes in differentiated 3T3-L1 adipocytes including GLUT-4 and PPARγ was considerably reduced after treatment with MCP-1 [57,58]. Although IL-6 is required for adipose-tissue macrophage accumulation, it causes weak changes in insulin sensitivity. FLRE showed an anti-lipolytic effect in TNF-α-induced adipocytes, whereas FLLE induced adipocyte lipolysis, which is consistent with the result of IL-6 expression, assuming that the increase in IL-6 by FLLE might induce lipolysis in adipocytes. It was previously known that IL-6 exerts a lipolytic effect but did not interfere with insulin sensitivity in adipocytes [59]. This could suggest that FLRE and FLLE might reduce inflammation in adipocytes and then suppress insulin resistance in TNF-α-induced adipocytes. Pro-inflammatory factors of adipocytes were activated via various pathways such as NF-κB and MAPK pathways, including ERK, JNK, and p38 [60]. FLRE and FLLE may exert their effects on adipocyte inflammation by different signaling pathways, which should be further investigated.

TNF-α has anti-adipogenic effects which down-regulate PPARγ and suppress other adipogenic genes, leading to loss of GLUT4 expression and insulin-stimulated activity [61]. PPARγ or CEBPα deficiency in adipocytes caused an absence of insulin-stimulated glucose transport by reducing tyrosine phosphorylation and expression of insulin receptors [62,63]. In our study, TNF-α inhibited mRNA expression of PPARγ and CEBPα, whereas FLRE and FLLE significantly improved their expressions, suggesting that the extracts abrogated TNF-α-interfered insulin signaling, reduced further inflammatory cytokine production and improved adipogenic gene expression, leading to the reduction in insulin resistance and dysfunction of adipocytes.

Adipocyte inflammation is related to macrophage recruitment and proinflammatory cytokine release from both cell types, leading to chronic inflammation surrounding adipose tissue [1]. As FLLE and FLRE showed anti-insulin resistance induced by TNF-α in adipocytes, the anti-inflammation ability of the extracts was further investigated in macrophages. FLRE, but not FLLE, significantly reduced NO production in LPS-induced RAW 264.7 macrophages. Our finding is similar to a previous study, which reported the anti-inflammation effect of *Ficus nota* (Blanco) Merr., which inhibited the production of NO depending on its total flavonoid content [64]. FLRE significantly decreased both iNOS mRNA and protein levels, which may contribute to the reduction in NO production. Moreover, COX-2 mRNA and protein, an inflammatory mediator which can be stimulated by NO [65], was significantly decreased by FLRE in LPS-induced macrophages. The inhibition of COX-2 expression may lead to the suppression of PGE2 production, which reduces the pathogenesis of inflammation such as pain and vasodilation [65] and prevents inflammation-induced diseases [66]. iNOS and COX-2 are stimulated via IKK and MAPK signaling by the group of pro-inflammatory cytokines including TNF- α, IL-1β, and IL-6 [67]. FLRE significantly decreased TNF-α (protein), IL-1β (mRNA) expression, and IL-6 (protein and mRNA) in LPS-induced macrophages. Likewise, *Ficus exasperata* Vahl significantly inhibited NO levels and inflammatory mediators (TNF-α and IL-1β) [68]. Additionally, *Ficus religiosa* Linn significantly inhibited the production of many inflammatory mediators (TNF-α, IL-1β, IL-6, iNOS, and COX-2) via down-regulation of MAPK and NF-κB signaling pathways [69]. Several data support the fact that ERK1/2 predominantly triggers the expression of IL-1β and IL-6, whereas JNK is the main transduction pathway of TNF-α, after LPS stimulation [70]. Furthermore, Fengying Huang et al. (2013) reported that IL-1β up-regulated the COX-2 expression through the activation of the p38 MAPK signaling pathway [71]. Therefore, it might be suggested that FLRE may inhibit LPS-stimulated signaling pathways via MAPK, leading to the reduction in inflammatory-related molecules.

In summary, we have shown that *Ficus lindsayana*, especially the root extracts, could effectively alleviate the inflammation-induced insulin resistance in 3T3-L1 adipocytes. Moreover, it can inhibit inflammation in both adipocytes and RAW 264.7 macrophages. These results may provide scientific data for the utilization and development of this plant as an alternative medicine or functional food for the prevention of inflammation and inflammation-related diseases including obesity, as well as type 2 diabetes mellitus.

## 4. Materials and Methods

All methods were performed in accordance with the relevant guidelines and regulations.

### 4.1. Chemicals and Reagents

TNF-alpha was purchased from PeproTech (Rehovot, Israel). Dexamethasone (Dex), 3-isobutyl-1-methylxanthine (IBMX), insulin, and lipopolysaccharide (LPS) were purchased from Sigma-Aldrich (St. Louis, MO, USA). All the primers for RT-qPCR were purchased from Bio Basic Inc., (Markham, ON, Canada). Anti-COX-2, anti-NF-κB, and anti-phospho-NF-κB p65 antibodies were obtained from Cell Signaling Technology (Danvers, MA, USA). Anti-iNOS and anti-β-actin antibodies were from Sigma-Aldrich (St. Louis, MO, USA). Clarity Western ECL Substrate was purchased from Bio-Rad Laboratories (Hercules, CA, USA).

### 4.2. Preparation of Plant Extracts

*Ficus lindsayana* collection was conducted following the guidelines and regulations of the legislation and deposited at Thailand Natural History Museum (THNHM, Bangkok, Thailand), Thailand Science Park, under a code of Chantarasuwan 040117-1. The dried samples of *Ficus lindsayana* root and latex were ground. The powder of *Ficus lindsayana* latex (FLL) was soaked with deionized water at room temperature in the ratio of 1:10 *w*/*v*. Then, the aqueous fraction was filtrated and freeze-dried by lyophilizer to obtain FLL extract (FLLE). The powder of *Ficus lindsayana* root (FLR) was soaked with 80% ethanol overnight at room temperature in the ratio of 1:10 *w*/*v* and this was repeated twice. The ethanolic extract was concentrated by a rotary evaporator and freeze-dried by lyophilizer, obtaining FLR extract (FLRE). The extracts were kept at −30 °C until use.

### 4.3. Phytochemical Analysis

The total flavonoid and total phenolic contents were determined by the colorimetric method using the aluminum chloride and the Folin–Ciocalteu reagent, respectively, published in the previous publications [72,73], with slight modifications. The catechin and apigenin equivalents in each extract were estimated as mg CE and APE/g extract, respectively, using a standard curve to represent total flavonoid content. Total phenolic content was presented as mg gallic acid (GAE) or chlorogenic acid (CGAE)/g extract, equivalent to the standard curves of gallic acid and chlorogenic acid, respectively.

HPLC was performed to analyze known phenolics [31] and chlorogenic acids (CGAs), including neochlorogenic acid (3-CQA), cryptochlorogenic acid (4-CQA), and chlorogenic acid (5-CQA) (IUPAC name) [74] existing in the FL extracts as previously described, with slight modification. The C18 column (250 mm × 4.6 mm, 5 µm) was used. Gradient elution was performed using two solvents: A (0.1% trifluoroacetic acid (TFA) in water) and B (100% methanol) for detection and determining phenolic compounds with a flow rate of 1.0 mL/min and detection at 280 and 325 nm. The CGA chromatographic separation was carried out using gradient mode with mobile phase A (0.3% TFA in water) and mobile phase B (100% acetonitrile) with a flow rate of 1 mL/min and detection at 280. The phytochemical contents were determined by using a gradient system of mobile phase A (1% acetic acid in water) and mobile phase B (100% acetonitrile) with a flow rate of 0.7 mL/min and detection at 280. The peak area and retention time of the extracted sample used in the present HPLC method were determined to compare with standard curves of various concentrations of standard apigenin, catechin, chlorogenic acid, coumaric acid, ferulic acid, gallic acid, hydroxybenzoic acid, protocatechuic acid, vanillic acid, 3-CQA,4-CQA, and 5-CQA.

Moreover, identification and quantification of phenolics were performed utilizing liquid chromatography–electrospray ionization tandem mass spectrometry (LC-ESI-MS/MS), with a well-established procedure and validation as previously reported [75,76].

### 4.4. Determination of Antioxidant Activities

The 2,2′-azinobis-(3-ethylbenzothiazoline-6-sulfonic acid) (ABTS) and the 2,2-diphenyl-1-picrylhydrazyl (DPPH) assays were performed according to the previous studies [77,78] with slight modifications to assess radical scavenging ability of the extracts. Using the following equation, both antioxidant activities were estimated as a % inhibition.

ABTS^•+^ or DPPH^•^ scavenging effect (%) = (Abs_control_ − Abs_extract_) × 100/Abs_control_

The radical scavenging activity of ABTS or DPPH was expressed as SC50, which represents the concentration of the extract (mg/mL) required to scavenge 50% of the radicals.

### 4.5. Cell Culture

Macrophage-like cells, RAW 264.7, were purchased from CLS-Cell Lines Service, Germany, and cultured in Dulbecco’s Modified Eagle Medium (DMEM, Sigma, St. Louis, MO, USA) supplemented with L-glutamine, 10% heat-inactivated fetal bovine serum (FBS, Sigma, St. Louis, MO, USA) and 1% penicillin/streptomycin solution (FBS, Sigma, St. Louis, MO, USA). The cells were maintained in a Corning^®^ 60 mm Ultra-low attachment culture dish at 37 °C in a 5% CO_2_ humidified atmosphere (CO_2_ incubator, Thermo Scientific, Waltham, MA, USA) and sub-cultured every three days. 

Human blood samples from healthy individuals (five samples) anonymized by the laboratory were taken from Maharaj Hospital, Chiang Mai, Thailand, and kept in a heparinized tube. Human peripheral blood mononuclear cells (PBMCs) were isolated using Ficoll–Hypaque separation, as directed in the manufacturer’s instructions [79]. The remained red blood cells (RBCs) were employed in a hemolysis test. This study was granted a certificate of exemption by the Research Ethics Committee of the Faculty of Medicine, Chiang Mai University (No. EXEMPTION 4744/2017). Consent to participate was not applicable because of anonymous data collection. All methods were carried out in accordance with relevant guidelines and regulations.

3T3-L1 adipocyte cells from ATCC (American Type Culture Collection) were cultured in DMEM with L-glutamine supplemented with 10% FBS and 1% penicillin/streptomycin solution and maintained at 37 °C in a 5% CO_2_ humidified atmosphere CO_2_ incubator. After reaching confluence on day 2, 3T3-L1 preadipocytes were differentiated by adding 0.5 mM 3-isobutyl-1-methylxanthine (IBMX), 0.5 µg/mL dexamethasone, 5 µg/mL insulin and 10% FBS for 72 h, followed by another 72 h in the same medium without IBMX and dexamethasone. Complete cell differentiation was obtained by incubating the cells in DMEM containing 10% FBS for six days.

### 4.6. Cytotoxicity

Cytotoxicity of FLLE and FLRE was examined in RAW 264.7 macrophages. The cells (25,000 cells/well) were plated into a 96-well cell culture plate and incubated for 24 h. The cells were then treated with different concentrations of FLLE or FLRE (0–800 µg/mL) for 48 h. Next, the cytotoxicity of the extract was determined by MTT assay as described elsewhere [80,81]. Briefly, MTT (0.5 mg/mL) in DMEM was added to each well. Then, the plates were further incubated for four hours and the medium was removed. Formazan crystals were dissolved with 100 μL of dimethyl sulfoxide (DMSO). The absorbance was measured at 560 nm and percentages of cell viability were calculated. 

Human peripheral blood mononuclear cells (PBMCs) were used to investigate the biosafety and cytotoxicity of the extracts for human immune cells. The isolated PBMCs were plated into a 96-well plate at the proper density (8 × 10^4^ cells/well) and incubated for 24 h. Next, the cells were treated with various concentrations of FLLE or FLRE (0–800 µg/mL) for 48 h. 

In 3T3-L1 adipocytes, the cells were plated in a 96-well plate (3000 cells/well) and incubated for two days. Then, the cells were differentiated by induction media, differentiation media, and maturation media. Until the cells became mature adipocytes, the cells were treated with various concentrations of FLLE or FLRE (0–200 µg/mL). 

The cytotoxicity of the extracts was determined in the PBMCs and mature adipocytes by SRB assay, as described previously [82]. The percent cell survival was calculated as follows.

% Cellular Viability = ((Abs of treatment group)/(Abs of control group)) ×100

The inhibitory concentration of 20% (IC20) or a non-toxic concentration was used for further experiments.

### 4.7. Hemolysis Assay

A hemolysis assay was used to determine the hemolytic effect of the FLLE and FLRE. In brief, human RBCs were collected after the separation of PBMCs from whole blood. The 5% RBCs suspension was then mixed with several concentrations of the extracts and incubated at 37 °C for 3 h. The hemolysis induction was measured as described previously [83]. The hemolysis guideline indicates that when <10%, hemolysis is non-hemolysis, and >25% hemolysis is a high hemolysis induction. 

### 4.8. Adipogenesis

To determine the effect of FLLE and FLRE on adipogenesis, the extracts at 50, 100, and 200 µg/mL were added during the adipocyte transformation process (days 1–12). Cells cultured with the vehicle (DMSO) in all three types of transformation medium were set as the untreated control.

After the treatment, the cells were then fixed in fresh 4% paraformaldehyde and the lipid droplets were stained with a 60% Oil Red O diluted in 100% 2-isopropanol, as previously described. The stained-intracellular lipid droplets were dissolved in 100% 2-isopropanol and the absorbance was measured by spectrometry at 520 nm (Gen5, BioTek). The experiment was performed in triplicate and repeated three times, independently.

### 4.9. Glucose Uptake Assay

Insulin resistance was induced in mature adipocytes by 50 ng/mL TNF-α and they were treated with or without the extract for 24 h. Then, the cells were washed with PBS and starved with low-glucose DMEM for 3 h. After washing, 1 mg/mL bovine serum albumin containing 100 mmol/L 2-NBDG-glucose and 50 nmol/L insulin was added to the cells and they were incubated at 37 °C for 1 h. The fluorescence intensity of intracellular 2-NBDG-glucose was measured by lysis of the cells with 90% DMSO and by placing the lysate in a dark place for fluorescence measurement (excitation at 485 nm and emission at 535 nm).

### 4.10. Lipolysis Assay

Adipocyte lipolysis was determined by measurement of released glycerol in a culture medium. The glycerol assay kit (Sigma Aldrich, St. Louis, MO, USA) was used following the instruction of the manufacturer. Afterward, insulin resistance was induced in mature adipocytes by 50 ng/mL TNF-α and they were treated with or without the extracts for 24 h; the culture supernatant was collected to determine free glycerol content. A total of 25 µL of each supernatant was added to 100 µL of Free Glycerol Reagent in a 96-well plate. The mixture was incubated for 15 min at room temperature. The absorbance was measured at 540 nm. The amount of glycerol was calculated using the equation of the standard curve in the formula below.

Free glycerol (µg/mL) = (Abs _540_ − (y-interception))/Slope

### 4.11. Measurement of Inflammatory Mediator and Adipogenic mRNA Expressions by Reverse Transcription–Quantitative Polymerase Chain Reaction (RT-qPCR)

After the treatment, total RNA was extracted from the cells and quantified by nanodrop spectrophotometer. Then, total RNA was reverse-transcribed into cDNA. mRNA level was measured by reverse transcription-quantitative polymerase chain reaction (RT-qPCR) using specific primers for TNF-α (sense, 5′-TCTCATGCACCACCATCAAGGACT-3′; antisense,5′-CCACTCTCCCTTTGCAGAACTCA-3′), iNOS (sense, 5′-TCTTTGACGCTCGGAACTGTAGCA-3′; antisense, 5′-ACCTGATGTTGCCATTGTTGGTGG-3′), COX-2 (sense, 5′-TACTACACCAGGGCCCTTCC-3′; antisense, 5′- CATATTTGAGCCTTGGGGGT-3′), IL-1β (sense, 5′-AAGGGCTGCTTCCCAACCTTTGAC-3′; antisense, 5′-ATACTGCCTGCCTGAAGCTCTTGT-3′), IL-6 (sense, 5′-ATCCAGTTGCCTTCTTGGGACTGA-3′; antisense, 5′-TAAGCCTCCGACTTGTGAAGTGGT-3′), MCP-1 (sense, 5′-CACTCACCTGCTGCTACTCA-3′; antisense, 5′-AGGGCCGGGGTATGTAACT-3′), CEBPα (sense, 5′-GCAAAGCCAAGAAGTCGGTG-3′; antisense, 5′- TCACTGGTCAACTCCAGCAC-3′), PPARγ (sense, 5′-TCCGCTGATGCACTGCCTAT-3′; antisense, 5′-GGAATGCGAGTGGTCTTCCA-3′), β-actin (sense, 5′-ACACCCCAGCCATGTACG-3′; antisense, 5′-TGGTGGTGAAGCTGTAGCC-3′), and GAPDH (sense, 5′-ACCACAGTCCATGCCATCAC-3′; antisense, 5′-TCCACCACCCTGTTGCTGTA-3′). The results of the target mRNA were relative to the housekeeping gene mRNA level, and the values obtained in the presence or absence of the extracts were expressed in relation to the values obtained in the control group.

### 4.12. Determination of Nitric Oxide Production

RAW 264.7 macrophages were plated in a 96-well plate and cultured for 24 h. The cells were then pretreated for 1 h with a non-toxic concentration of FLLE or FLRE (0–200 g/mL). After that, 1 µg/mL LPS was added to each well and incubated for 24 h. The culture supernatant was then collected to investigate NO generation using Griess reagent assay. Griess reagent and supernatant were mixed and incubated for 15 min at room temperature, in the dark. The absorbance was measured at 540 nm. In addition, the treated cells were subjected to MTT assay for the determination of the cell viability.

### 4.13. Enzyme-Linked Immunosorbent (ELISA) Assay

TNF-α or IL-6 levels secreted from the treated cells to the culture media were assessed using a sandwich Enzyme Link Immuno-Sorbent Assay performed according to manufacturers’ guidelines (BioLegend’s ELISA MAXTM Deluxe Set, San Diego, CA, USA).

### 4.14. Western Blotting

The treated cells were collected to prepare protein samples. The samples were subjected to 10% SDS-PAGE, and the proteins were electrically transferred to a nitrocellulose membrane. The target proteins were probed with specific antibodies (anti-COX-2 antibody (1:2000), anti-iNOS antibody (1:2000), anti- β-actin antibody (1:10,000), anti-NF-κB antibody (1:1000), and anti-phospho-NF-κB p65 (1:1000)). The enhanced chemiluminescence (ECL) method was used for the protein detection. The iNOS, COX-2, and β-actin visible bands were 131, 74, and 42 kDa, respectively.

### 4.15. Statistical Analysis

All values are reported as mean ± standard derivation (X ± SD) of three independent tests using triplicate samples. The one-way analysis of variance (ANOVA), followed by Tukey’s multiple comparison tests was employed using Prism 9.4.1 software to determine overall differences among the treatment groups. Statistical significance is noted when the *p* values < 0.05.

## 5. Conclusions

In conclusion, FLLE and FLRE showed adipogenesis induction ability by increasing lipid accumulation, suggesting that the extracts might prevent hypertrophic adipocytes, leading to the reduction in inflammation, dysfunction, and insulin resistance of the cells. Moreover, FLLE and FLRE could improve insulin sensitivity in TNF-α-induced adipocytes via the decrease in MCP-1 expression, which may reduce inflammatory cascades and improve adipocyte function. Furthermore, FLRE exhibited anti-inflammation activity via the suppression of inflammation-related molecule expression. The extracts were less toxic, as determined by in vitro cytotoxic assays. Finally, the knowledge from the present study provides scientific data and useful information on the biological potential of *Ficus lindsayana*. However, identification of bioactive compounds and in vivo safety assessment must be performed for further development and use as either a herbal medicine or a functional ingredient.

## Figures and Tables

**Table 1:** Total Phenolic and Total Flavonoid Contents and DPPH or ABTS Free-Radical Scavenging Activities (SC50) of FLLE and FLRE.

FL Extracts	Total Phenolic (mg GAE/g Extract)	Total Flavonoid (mg CE/g Extract)	DPPH Assay SC50 ^1^ (µg/mL)	ABTS Assay SC50 (µg/mL)
FLLE	248.53 ± 1.46 **	55.78 ± 4.00	210.59 ± 6.13	33.59 ± 6.26
FLRE	208.31 ± 9.90	157.33 ± 1.36 ***	83.12 ± 8.86 ***	9.44 ± 3.26 **

^1^ SC50 = the concentration of the extracts that scavenge 50% of DPPH or ABTS free radicals. FL, *Ficus lindsayana*; GAE, gallic acid equivalent; CE, catechin equivalent. Each value is represented as mean ± SD, *n* = 3 ** p < 0.01 and *** p < 0.001 in FLRE vs FLLE.

**Table 2:** Total Phenolic Contents Using Chlorogenic Acid and Total Flavonoid Contents Using Apigenin Equivalent and Quantification of Chlorogenic Acid Derivatives and Apigenin Using HPLC in FLLE and FLRE.

			Chlorogenic Acid (CGAs) (mg/g Extract)			Apigenin (AP) (mg/g Extract)
FL Extracts	Total Phenolic (mg CGAE/g Extract)	Total Flavonoid (mg APE/g Extract)	3-CQA	4-CQA	5-CQA	Apigenin
FLLE	517.28 ± 2.72	33.55 ± 1.20	39.85 ± 0.88	19.80 ± 0.52	26.00 ± 0.93	ND ^1^
FLRE	111.96 ± 0.67	51.81 ± 2.88	4.98 ± 0.10	1.08 ± 0.06	0.46 ± 0.08	0.93 ± 0.09

FL, *Ficus lindsayana*; CGAE, chlorogenic acid equivalent; APE, apigenin equivalent; 3-CQA, neochlorogenic acid; 4-CQA, cryptochlorogenic acid; 5-CQA, chlorogenic acid. Each value is represented as mean ± SD, *n* = 3. ^1^ ND not detectable.

**Table 3:** Quantification of Phenolic Contents Using LC-MS/MS.

	FLLE	FLRE
Compounds	mg/g Extract	Mg/G Extract
Gallic acid	ND ^1^	0.08
Chlorogenic acid (5-CQA)	3.34	0.74
3,4-Dihydroxybenzoic acid	ND	0.56
Caffeic acid	0.40	0.21
Luteolin	ND	0.24
Genistein	ND	1.40
Apigenin	ND	0.88
Naringenin	ND	0.53

^1^ ND not detectable.

**Table 4:** Hemolysis Effect of FLLE and FLRE.

Extracts (µg/mL)	% Hemolysis (*n* = 5)
0.05% Triton x-100 ^1^	100 ± 0.00
FLLE	
200	4.55 ± 0.70
400	4.90 ± 0.79
600	5.41 ± 0.73
800	5.95 ± 0.65
FLRE	
200	5.59 ± 0.43
400	8.12 ± 1.36
600	10.70 ± 2.22
800	12.70 ± 3.19

^1^ 0.05% triton x-100 was used as a positive control. The results were expressed as mean ± SD, *n* = 5 (hemolysis, (0–10% hemolysis is non-hemolysis, 10–25% hemolysis is a slight hemolysis induction, and >25% hemolysis is a high hemolysis induction.).

## Data Availability

The datasets used and/or analyzed during the current study are available from the corresponding author on reasonable request.
